# Manual Therapy in Post-operative Knee Management: A Systematic Review of Effects on Pain in Total Knee Replacement (TKR) Patients

**DOI:** 10.7759/cureus.92734

**Published:** 2025-09-19

**Authors:** Sidharth Sahni, Joseph E Fares, Harrison Jordan

**Affiliations:** 1 Physical Medicine and Rehabilitation, New York University, New York, USA; 2 Physical Medicine and Rehabilitation, Inspira Health Network, Mullica Hill, USA; 3 Physical Medicine and Rehabilitation, East Carolina University, Greenville, USA

**Keywords:** functional recovery, kinesiotaping, manual lymphatic drainage, manual therapy, osteopathic manipulative treatment, pain management, postoperative pain, randomized controlled trials, rehabilitation, total knee replacement

## Abstract

Manual therapy is a hands-on treatment approach commonly used by healthcare professionals, such as doctors of osteopathic medicine, physical therapists, chiropractors, and massage therapists, to address musculoskeletal pain and improve functional recovery. Although its effectiveness is well established for various musculoskeletal conditions, its role in postoperative knee rehabilitation, particularly after total knee replacement (TKR), remains underexplored. Postoperative pain following TKR can significantly affect mobility and delay recovery, underscoring the need for effective rehabilitation strategies. While individual studies have reported mixed outcomes of manual therapy after TKR, the evidence base is fragmented and lacks consensus. A systematic review is therefore warranted to synthesize current findings, evaluate the quality of available evidence, and clarify whether manual therapy provides meaningful benefits in pain reduction, functional outcomes, or patient satisfaction after TKR.

The primary objective of this study was to review existing randomized controlled trials (RCTs) to evaluate the impact of manual therapy on pain management in patients following TKR surgery. The study aimed to synthesize current evidence regarding the effectiveness of different manual therapy techniques in reducing postoperative pain and enhancing functional outcomes.

A comprehensive literature search was conducted using multiple databases, including PubMed, CINAHL, Cochrane, and Ovid Medline. The initial search yielded 332 articles, which were screened against predefined inclusion and exclusion criteria. After screening, 10 RCTs focusing on manual therapy as the primary intervention in postoperative knee patients were selected for detailed review. The interventions studied included osteopathic manipulative treatment, manual lymphatic drainage (MLD), Kinesio Taping (KT), and multimodal manual therapy techniques. Data regarding pain outcomes, treatment protocols, and follow-up durations were extracted and analyzed.

The reviewed studies suggest that manual therapy can effectively reduce pain, improve joint mobility, and decrease the use of pain medications during the early postoperative period. MLD and KT showed particular promise in providing short-term pain relief and reducing swelling when applied soon after surgery. Some studies also reported improved patient satisfaction and faster functional recovery with manual therapy. However, findings were inconsistent across studies due to differences in treatment timing, methodologies, and patient populations. Long-term benefits of manual therapy remain unclear, emphasizing the need for further research.

Manual therapy appears to be a viable complementary treatment option for managing pain and improving functional recovery after TKR surgery. Its hands-on nature, lack of specialized equipment requirements, and adaptability to individual patient needs make it an attractive rehabilitation approach. Despite encouraging preliminary evidence, more rigorous research, with larger sample sizes and standardized protocols, is necessary to definitively establish the effectiveness of manual therapy and to guide clinical practice.

## Introduction and background

Manual therapy refers to a hands-on physical treatment provided by qualified healthcare professionals to address musculoskeletal pain, post-surgical discomfort, promote healing, and enhance mobility [1]. For the purposes of this review, manual therapy includes techniques such as osteopathic manipulative treatment (OMT), high-velocity low-amplitude (HVLA), manual lymphatic drainage (MLD), myofascial release, Kinesio Taping (KT), and soft tissue mobilization. These approaches are commonly used by providers such as doctors of osteopathic medicine (DO), physical therapists (PTs), chiropractors, and massage therapists [2].

While the effectiveness of manual therapy in treating conditions like low back and neck pain is well documented, its use in postoperative knee rehabilitation, particularly after total knee replacement (TKR), is less established. Much of the existing research has focused on manipulation under anesthesia (MUA) or on non-surgical musculoskeletal conditions, leaving a gap in understanding about the role of manual therapy after surgery.

Given the global use of manual therapy and the ongoing challenge of managing pain and restoring function after TKR, this review seeks to evaluate the current evidence on the impact of manual therapy techniques on pain outcomes in patients recovering from knee replacement.

The knee is a uniaxial joint that primarily moves in one plane, but also permits some rotational movement. It is supported by intra-articular structures such as the menisci, articular cartilage, anterior cruciate ligament, posterior cruciate ligament, lateral collateral ligament, and medial collateral ligament, all of which allow the knee to maintain a full range of motion (ROM) and stability. The medial and lateral menisci cushion the joint, but this arrangement also makes the knee highly vulnerable to injury [3,4].

TKR is a common surgical intervention for individuals with severe knee joint damage, typically from advanced osteoarthritis or traumatic injury. When conservative management fails, TKR replaces the damaged joint surfaces with prosthetic components to restore function and relieve pain [5]. Despite these benefits, many patients experience significant postoperative pain that can impair mobility and slow recovery. Early improvements in function and pain relief after TKR are predictive of long-term success, making effective rehabilitation critical [6-8]. Persistent pain highlights the need for adjunctive strategies, and manual therapy has been proposed as a hands-on intervention that may enhance functional outcomes and pain management in this context.

Manual therapy has a long history, dating back to 400 BCE [9], and has since been refined into structured approaches delivered by trained professionals, including DOs, PTs, chiropractors, and massage therapists. DOs employ osteopathic manipulative medicine (OMM) to address musculoskeletal dysfunction and other conditions, often integrating it with medical therapy to improve outcomes and even reduce the need for pain medication [10,11]. PTs frequently use manual therapy to restore mobility, improve function, reduce pain, and prevent complications such as deep vein thrombosis in post-surgical patients [3,12-14]. Chiropractors contribute through joint mobilizations, extremity adjustments, and myofascial techniques that restore biomechanical balance, reduce inflammation, and improve post-surgical recovery [2,15,16]. Massage therapists also play a role, using soft tissue manipulation to reduce stress, control pain, and promote recovery after surgery, making it a safe and cost-effective adjunct to other therapies [17,18].

OMT encompasses a wide range of manual therapy techniques designed to address structural and functional imbalances in the body. Common modalities under this umbrella include MLD, KT, myofascial release, instrument-assisted soft tissue mobilization, balanced ligamentous tension (BLT), deep friction massage, manual trigger point therapy, soft tissue mobilization, muscle energy technique (MET), HVLA thrust, and counterstrain. Core OMT techniques such as HVLA, MET, myofascial release, lymphatic pump, counterstrain, traction, and craniosacral therapy reflect the diversity and adaptability of this hands-on approach [19]. By utilizing a combination of the aforementioned techniques, manual therapy can play a significant role in enhancing ROM, reducing pain, and accelerating recovery in various musculoskeletal conditions, including during post-surgical rehabilitation [20]. Manual therapy has also been shown to alleviate pain by activating pain-modulating pathways, reducing muscle spasms, and improving circulation to injured areas [1]. As a result, many patients experience reduced discomfort and enhanced functional ability, often leading to faster recovery times compared to those who rely solely on exercise or other conservative interventions. While the application of manual therapy in knee rehabilitation has yet to be fully explored, preliminary findings suggest that it may facilitate quicker recovery, improve joint function, and reduce reliance on pain medication, thereby offering a promising complement to traditional rehabilitation protocols [21].

## Review

Materials and methods

The purpose of this study was to review the literature pertaining to manual therapy’s effect on pain in postoperative knee patients.

Database

The databases used for this study were PubMed, CINAHL, Cochrane, and Ovid Medline. Certain journal titles include JAOA, Journal of Arthroplasty, Alternative Therapies, JOSPT, International Journal of Therapeutic Massage and Bodywork, Journal of Manual and Manipulative Therapy, Archives of Physical Medicine and Rehabilitation (ACRM), Journal of Bodywork and Movement Therapies, and the Journal of Knee Surgeries.

Search Terms

The search terms used to find current studies were: "knee joint", "arthroplasty, replacement", "knee injuries", "manipulation", "osteopathic", "pain", "NPRS", "NPR", "NRS", "VAS", "musculoskeletal manipulation", "osteopathic treatment", "chiropractic", "chiropractor", "physical therapy", "manual therapy", "joint mobilization", "ligament injury", "manual lymphatic drainage" (MLD), "myofascial release", "instrument-assisted soft tissue mobilization" (IASTM), "massage therapy", "balanced ligamentous tension", "counterstrain", "muscle energy technique" (MET), "ligamentous articular strain", "soft tissue mobilization", "deep friction massage", "Graston", "manual trigger points", and "HVLA". Combinations of these search terms were used to retrieve a total of 332 articles.

Inclusion and Exclusion Criteria

The inclusion criteria selected articles meeting all of the following requirements: postoperative knees; manual treatment was the primary intervention and focus of the study; studies tracked pain as an outcome; studies were randomized controlled trials (RCTs); treatment provided by a licensed healthcare professional, including but not limited to PTs, chiropractors, osteopathic physicians, or massage therapists; available in English; and included human participants. Selection criteria also specifically excluded articles that did not include a medical complication of the knee; were not related to postoperative patients; focused on arthritis, non-surgical related diagnoses, acupuncture, continuous passive motion (CPM), MUA, or manipulation not related to the knee joint and/or categories of similar nature; articles that were not peer reviewed; articles that did not focus on manual therapy as a primary intervention; and those that did not use outcome measures to track the benefits of manual treatment.

Data Extraction and Quality Assessment

The initial articles were screened for eligibility by four independent reviewers and then reassessed by two of the same reviewers to confirm inclusion. To ensure the review was up to date, a final search was repeated by an additional reviewer, who also served as a tiebreaker if consensus could not be reached. Sample sizes varied among the included studies, which should be considered given their effect on comparability. Risk of bias for the included RCTs was assessed independently by two reviewers using the Cochrane Risk of Bias Tool for Randomized Trials (RoB 2) [22]. Domains evaluated included bias arising from the randomization process, deviations from intended interventions, missing outcome data, measurement of the outcome, and selection of the reported result. Any disagreements between reviewers were resolved through discussion or adjudication by a third reviewer.

In addition, the strength of evidence for each study was graded using the Oxford Centre for Evidence-Based Medicine (OCEBM) Levels of Evidence framework. In this system, Level 1B evidence designates an individual RCT with a narrow confidence interval, indicating high precision and reliability of the reported effect size. Classification was based on study design and statistical reporting; studies identified as RCTs with clearly reported, narrow 95% confidence intervals around their primary outcomes were designated as Level 1B. Two reviewers independently assigned levels of evidence, and discrepancies were resolved through consensus. This approach provides transparency and ensures the grading process can be replicated by future researchers.

Results

Search Result

**Figure 1:**
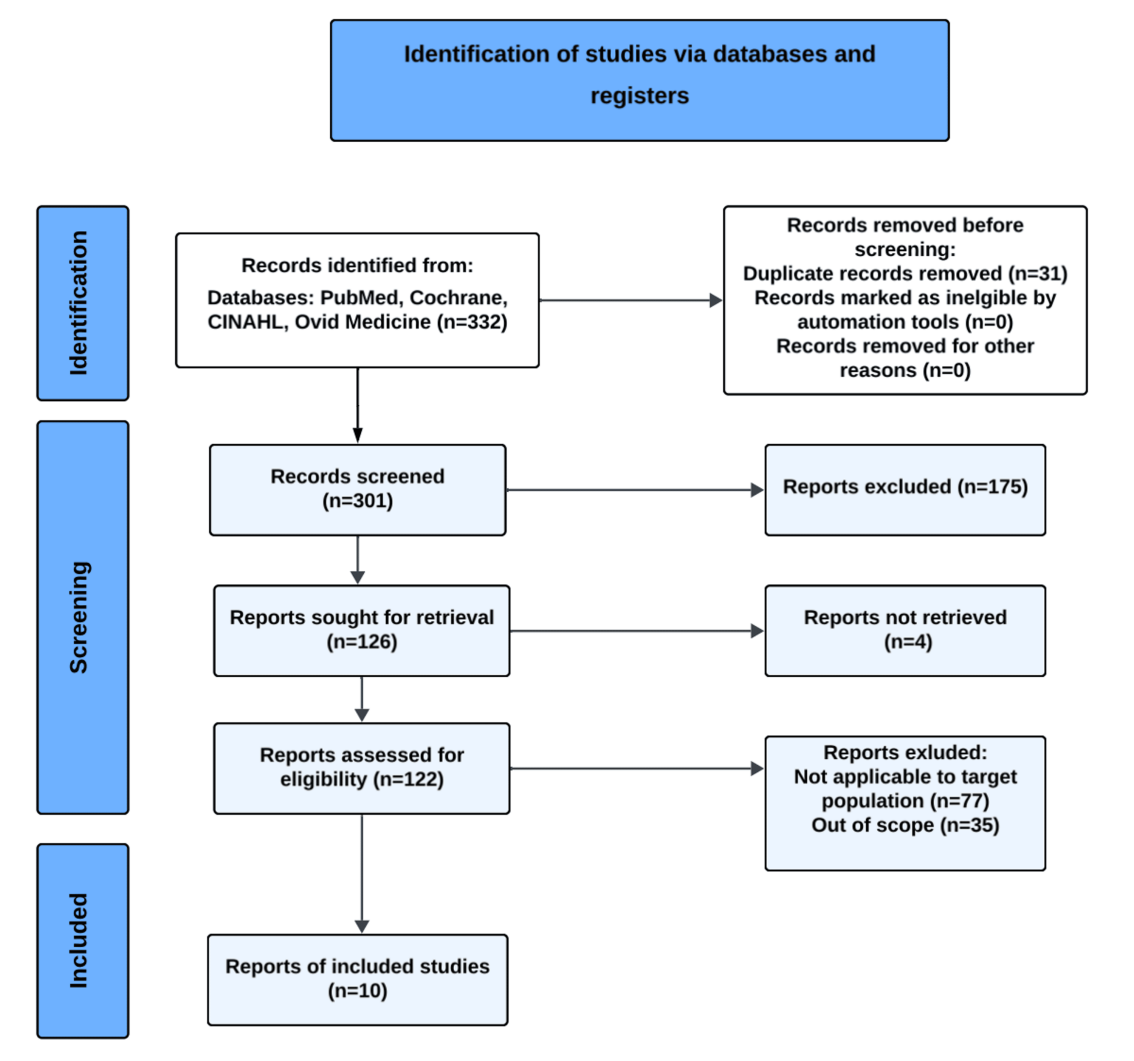
PRISMA Study Selection Process for Manual Therapy in Postoperative Total Knee Replacement Rehabilitation Adapted from Page et al. [23]

Our initial search yielded 332 articles, of which only 126 were reviewed due to most articles not meeting the inclusion and exclusion criteria. The final results yielded 10 articles that matched the criteria. The PRISMA flow diagram can be visualized in Figure 1.

The study selection pathway for our systematic review is illustrated in Figure 1, which presents the PRISMA flow diagram depicting the number of records identified, screened, excluded (with reasons), and the final RCTs included for analysis of manual therapy in postoperative TKR rehabilitation.

The researchers reviewed each study and collected data on the author, study population, outcomes utilized, treatment follow-up length, and overall conclusions regarding the effectiveness of interventions on pain reduction. Table 1 summarizes these findings [21,24-32], providing a detailed overview of the RCTs included in this review, including study populations, manual therapy interventions, comparator groups, and primary outcomes related to pain management following TKR surgery.

**Table 1:** Summary of Randomized Controlled Trials Evaluating Manual Therapy Interventions for Pain Management Following Total Knee Replacement Surgery Level 1B indicates an individual RCT with a narrow confidence interval. Statistical significance was defined as p < 0.05, as reported by the individual study authors. “Yes” indicates that the primary outcome met statistical significance; “No” indicates that it did not. Exact p-values were not uniformly reported across studies. Abbreviations: NPRS, Numeric Pain Rating Scale; VAS, Visual Analog Scale; NRS, Numeric Rating Scale; PT, Physical Therapy/Physical Therapist; MLD, Manual Lymphatic Drainage; OMT, Osteopathic Manipulative Treatment; HVLA, High-Velocity Low-Amplitude; ME, Muscle Energy; CPM, Continuous Passive Motion; IM, Intramuscular; RCT, Randomized Controlled Trial; TKA, Total Knee Arthroplasty; THA, Total Hip Arthroplasty; KT, Kinesio Taping

Author	Level of Evidence	Participants	Outcome Measured	Follow-up	Control	Intervention	Findings	Pain Outcome	Statistical Significance	Additional Notes
Karaborklu Argut et al. (2021) [24]	1B	42	NPRS	Two months post-surgery	Standard PT without Manual Therapy (N = 21)	Standard PT with Mobilization (patellofemoral and tibiofemoral joint glides, friction massages, and soft tissue mobilization) (N = 21)	Improvements in pain outcomes were significantly higher in the mobilization group, with statistically significant findings. The mobilization group also had higher patient satisfaction	Improved pain outcomes	Yes	Also had higher patient satisfaction
Pichonnaz et al. (2016) [21]	1B	53	VAS	Days 2, 7, and 3-months post-surgery	Standard PT with placebo treatment (five 30-minute tape-recorded relaxation sessions based on Ericksonian hypnosis and autogenic training) (N = 24)	Standard PT with 5 MLD treatments, administered between day 2 and day 7 (N = 29)	MLD has a transient pain-reducing effect immediately after treatment. This finding was statistically significant after 4 out of 5 MLD treatments	Transient pain reduction immediately after treatment	Yes (after 4 out of 5 treatments)	-
Ebert et al. (2013) [25]	1B	43	NPRS	Days 2-4, and 6-weeks post-surgery	Standard PT without manual therapy (N = 24)	Standard PT with MLD on days 2, 3, and 4 postoperatively (N = 26)	Using MLD showed no significant differences in reported pain. Lower NRS scores were noted overall in the MLD group; however, the data were not statistically significant	Lower NRS overall	No	Not statistically significant
Licciardone et al. (2004) (TKA + THA) [26]	1B	60	Medication dose change (acetaminophen and/or hydrocodone dosage changes)	4 weeks after discharge from rehab	Standard PT with Sham Treatment (of range-of-motion activities and light touch at substantially decreased magnitude and were purposely aimed at avoiding key areas of somatic dysfunction) (N = 30)	Standard PT with OMT (HVLA, ME, myofascial, counterstrain, soft tissue, craniosacral) (N = 30)	The OMT protocol did not amount to better pain control. Did see a decrease in analgesic medication (acetaminophen, hydrocodone), but no statistically significant data	No improvement in pain	No	Decrease in analgesic medication (acetaminophen, hydrocodone), not statistically significant
Jarski et al. (2000) (multiple dx) [27]	1B	39	Medication dose change (supplemental intramuscular analgesic use)	Days 2-5 post-surgery	Standard PT without manual therapy (N = 18)	Standard PT with OMT (HVLA, ME, myofascial, lymphatic drainage, counterstrain, traction) (N = 21)	The OMT group did not experience decreased pain. However, the OMT group required less supplemental IM analgesia; the difference was not statistically significant	No improvement in pain	No	Required less supplemental IM analgesia, but the difference was not statistically significant
Guney-Deniz et al. (2023) [28]	1B	45	VAS	Days 2-4, 2 weeks, and 6 weeks post-surgery	Standard PT without manual therapy (N = 15)	Standard PT with MLD treatment (n = 15) or standard PT with KT (n = 15)	Both the MLD and KT groups had lower pain levels on day 4 post-surgery. The beneficial effects lasted only 2 weeks. No group differences were found at 6 weeks	Lower pain on day 4 post-surgery	No (at 6 weeks)	Effects lasted only 2 weeks
Fujiura et al. (2020) [29]	1B	41	VAS	Day 10 post-surgery	Standard PT with manual therapy (includes skin and muscle mobilization) (N = 20)	Standard PT with manual therapy (skin and muscle mobilization) and once-daily MLD for 20 minutes prior to therapy for 5 sessions up to 10 days after TKA (N = 21)	Using MLD showed no significant difference in resting pain on day 10. Lower VAS scores were noted in the overall MLD group, but the data were not statistically significant. It was also noted that the intensity of pain at rest before and after MLD was significantly attenuated during interventions 1 and 3	No significant difference in resting pain on day 10	No (for overall group)/Yes (interventions 1 & 3)	Pain at rest significantly attenuated during interventions 1 and 3
Vergili et al. (2022) [30]	1B	16	VAS	Days 2, 4, and 6 post-surgery	Standard PT without manual therapy (N = 8)	Standard PT with MLD therapy on day 2 and day 4 postoperatively, using a 30-minute protocol (N = 8)	Using MLD had no significant difference in reporting daytime and nighttime pain	No significant difference in daytime or nighttime pain	No	-
Cihan et al. (2021) [31]	1B	21	VAS	Day 3 and 6 weeks post-surgery	Standard PT (including CPM) (N = 10)	Standard PT (including CPM) and MLD in the first 3 days post surgery (N = 11)	MLD had a significant effect in lowering pain on D3 and 6 weeks post-surgery	Lower pain on day 3 and at 6 weeks post-surgery	Yes	-
Tornatore et al. (2020) [32]	1B	99	NRS	Days 2, 4, and 6 post-surgery	None	Standard PT (including CPM) with KT and lymphatic therapy (n = 33), lymphatic drainage alone (n = 33), or KT alone (n = 33)	Both KT and MLD significantly reduced pain. A significantly higher improvement was observed in the KT and MLD combination group than in the individual group. No statistical difference between KT and MLD	Significant pain reduction	Yes	KT + MLD group had greater improvement than the individual groups; no significant difference between the KT & MLD

**Figure 2:**
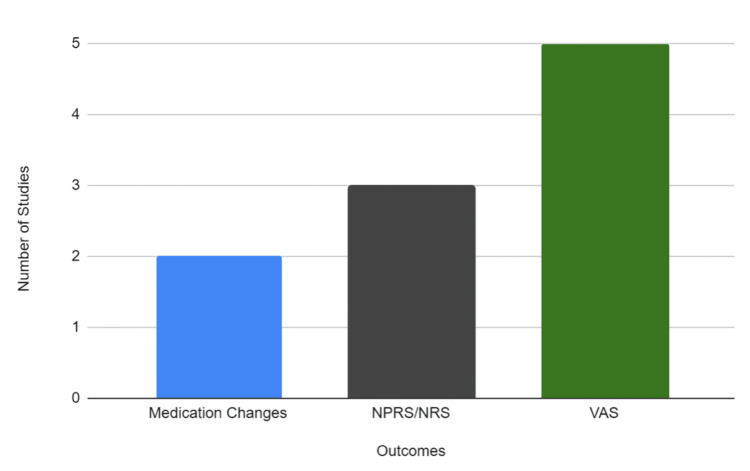
Outcome Measures Used to Assess Pain in Randomized Controlled Trials of Manual Therapy Following Total Knee Replacement Abbreviations: NRS, Numeric Rating Scale; NPRS, Numeric Pain Rating Scale; VAS, Visual Analog Scale

Figure 2 illustrates the range of outcome measures employed across the RCTs, demonstrating the different instruments and scales used to assess pain following manual therapy interventions in patients undergoing TKR.

Table 2 summarizes the specific manual therapy interventions evaluated in the included RCTs and identifies the corresponding studies, allowing for comparison of techniques and their application in postoperative TKR rehabilitation.

**Table 2:** Summary of Manual Therapy Interventions and Corresponding Studies Included in the Review *Fujiura et al. (2020) compared multimodal manual treatment alone to multimodal manual treatment combined with manual lymphatic drainage (MLD), and is therefore listed under both intervention categories [29]. **Guney-Deniz et al. (2023) assigned participants to one of three groups: standard exercise alone, exercise with MLD, or exercise with MLD and Kinesio Taping (n = 15) [28]. ***Tornatore et al. (2020) included three intervention groups: MLD with Kinesio Taping, MLD alone, and Kinesio Taping alone [32]. All participants underwent standard rehabilitation protocols, and assessments were conducted on postoperative days 2 through 4.

Interventions	Articles
Osteopathic Manipulative Treatment	Licciardone et al. (2004) [26]; Jarski et al. (2000) [27]
Manual Lymphatic Drainage	Cihan et al. (2021) [31]; Ebert et al. (2013) [25]; *Fujiura et al. 2020 [29]; **Guney-Deniz et al. (2023) [28]; Pichonnaz et al. (2016) [21]; ***Tornatore et al. (2020) [32]; Vergili et al. (2022) [30]
Physical Therapy Multimodal Manual Treatment	*Fujiura et al. (2020) [29]; Karaborklu Argut et al. (2021) [24]
Kinesio Taping	**Guney-Deniz et al. (2023) [28]; ***Tornatore et al. (2020) [32]

Discussion

Manual therapy has long been recognized as a key component of postoperative knee rehabilitation. This study aims to evaluate the effectiveness of manual therapy as a treatment option for pain management in patients following knee surgery. Previous research has demonstrated that manual therapy can be effectively administered by PTs [29], with additional benefits noted when performed by OMM practitioners, chiropractors, and massage therapists [26]. Our findings align with these studies, suggesting that manual therapy can be a valuable adjunct in treating postoperative knee patients. To our knowledge, this is the first study to explore the effects of manual therapy performed by all formally trained healthcare providers on postoperative knee patients and their relationship to pain reduction.

Manual therapy encompasses hands-on techniques delivered by various healthcare professionals. The knee, prone to frequent injuries, often requires surgical intervention; however, surgery can lead to postoperative pain, complications, and added financial burdens. Manual therapies have shown promise in reducing pain following knee replacement, with techniques aimed at improving joint mobility, reducing muscle tension, and enhancing circulation - all of which can alleviate discomfort and support recovery [18]. Despite its potential, there is a lack of published research specifically addressing manual therapy for postoperative knee rehabilitation, highlighting the need for further exploration in this area. Therefore, the goal of this study was to review existing literature on the application of manual therapy in postoperative knee care. To ensure the reliability of our findings, we conducted comprehensive searches, yielding 332 relevant articles. Articles that did not meet the inclusion criteria were excluded, and the remaining studies were categorized and assessed for their level of evidence, yielding a final total of 10 reported studies.

Included studies were RCTs that incorporated some form of manual therapy as part of their intervention and tracked changes in pain post-intervention. We ultimately included 10 RCTs in our analysis and compared and contrasted findings between these studies to assess the impact of these techniques.

The effectiveness of various rehabilitation techniques, including mobilization, MLD, KT, and OMT, in managing pain following TKR has been a subject of considerable investigation. These interventions, while often beneficial in clinical practice, yield mixed results across different studies, thus revealing the complexity of postoperative pain management and recovery outcomes.

Mobilization and OMT: Impact on Function and Analgesia

Karaborklu Argut et al. found that pairing a structured exercise program with manual therapy led to greater pain reduction compared to standard rehabilitation [24]. Patients in the mobilization group also reported higher levels of satisfaction with their care. In contrast, Licciardone et al.’s study showed no statistically significant difference in pain scores between those treated with OMT and those who received sham therapy, even after accounting for baseline differences [26]. Still, a reduction in the use of analgesic medications was observed in the OMT group, suggesting potential benefits beyond what pain scores alone reflect. Supporting this, Jarski et al.’s research showed that patients receiving OMT early in the postoperative period regained mobility more quickly, as they walked longer distances on days 1, 2, and 4, climbed stairs earlier, and were discharged sooner than the control group [27]. Like Licciardone et al.’s study, Jarski et al. also noted a decrease in pain medication use, underscoring OMT’s possible role in promoting recovery and limiting pharmaceutical reliance, even in the absence of significant changes in reported pain levels.

MLD and Its Efficacy

The impact of MLD on postoperative pain has been examined in several studies, with evidence largely pointing to short-term gains. Pichonnaz et al. reported that five additional MLD sessions reduced early-stage pain, with visual analog scale (VAS) scores dropping between 5.8 mm and 8.2 mm immediately following treatment [21]. These additional sessions produced statistically significant results, suggesting potential benefits for facilitating knee mobilization and early ambulation in patients experiencing acute discomfort. Similarly, Cihan et al. observed significant reductions in pain as early as the third day post-surgery, with effects lasting through the sixth week [31]. These improvements were also linked to reduced kinesiophobia and better overall quality of life, reinforcing MLD’s potential role in early recovery.

By contrast, other studies have reported less consistent results. Fujiura et al. found only a temporary reduction in resting pain after the first and third treatments, with no meaningful differences between MLD and control groups in long-term pain, swelling, strength, or function [29]. Lower VAS scores were noted in the overall MLD group, but the data were not statistically significant. Vergili et al. also noted a modest decrease in swelling by postoperative day 6 in the MLD group, but this did not correspond to improvements in pain or ROM [30]. Likewise, Ebert et al. observed no statistically significant differences between groups in pain outcomes using either the NRS or KOOS (Knee Injury and Osteoarthritis Outcome Score) subscale [25]. Lower NRS scores were noted overall in the MLD group; however, the data were not statistically significant.

Collectively, these findings highlight variability in MLD’s effectiveness and underscore the need for further research to clarify its role in TKA recovery.

Combined Therapies: MLD and KT

Some research has also explored the use of MLD alongside other modalities. Both Tornatore et al. and Guney-Deniz et al. investigated how MLD and KT might work individually or in combination to address post-surgical pain and edema [28,32]. In Guney-Deniz et al.'s study, patients who received either MLD or KT, in addition to standard exercises, experienced less pain by postoperative day 4, with benefits lasting through the second week. However, by six weeks, the differences between groups had largely disappeared. Tornatore et al. compared three approaches that included either MLD alone, KT alone, or both combined. Patients receiving the combination showed the most improvement during the first week, particularly on day 1. Though the study didn’t follow patients beyond day 6 and lacked a control group, the early outcomes suggest a possible additive effect when both techniques are used together.

Taken together, these studies indicate that MLD and KT can be effective tools in managing acute pain and swelling, especially when applied early. Their low cost and ease of implementation also make them appealing options to support standard rehabilitation in the immediate postoperative period.

Contrasting Findings and Considerations for Future Research

While studies such as those by Pichonnaz et al. and Cihan et al. highlight MLD’s potential to reduce pain and aid recovery in the short term, the broader body of evidence remains mixed [21,31]. Findings from Fujiura et al., Vergili et al., and Ebert et al. suggest that MLD may have limited long-term effectiveness, with no significant differences in overall pain scores or swelling when compared to control groups [25,29,30]. However, Fujiura et al.’s study did report transient reductions in resting pain after early interventions, as well as a modest improvement in knee extension at three months, indicating some benefit during the recovery process. Given the variability in study design, treatment protocols, and outcome measures, further research is needed to determine which patients are most likely to benefit from MLD and at what stage of recovery the intervention is most effective. As Vergili et al. noted, larger sample sizes may be necessary to better assess the significance of these findings, but early intervention appears promising.

Moreover, the utilization of MLD alone or with KT may offer a more holistic approach to rehabilitation. Tornatore et al. and Guney-Deniz et al.’s findings suggest that these therapies can enhance pain relief and edema reduction, especially during the early postoperative period [28,32]. These short-term improvements may help with pain tolerance, which can facilitate mobilization, stair negotiation, and gait training, overall helping with key milestones in early TKA recovery. While KT and MLD may not offer sustained benefits beyond the initial weeks, they can provide a window of reduced discomfort that allows patients to engage more fully in physical therapy and regain function.

In conclusion, while mobilization, OMT, KT, and MLD each show some degree of efficacy in improving pain and function after TKA, the inconsistency in results underscores the complexity of managing postoperative pain. These modalities, particularly when applied acutely, may offer a temporary but meaningful reduction in discomfort, and can possibly decrease the need for analgesics and encourage earlier participation in rehabilitation. Although the effects may not last, even short-term relief can provide patients with momentum during the critical early days of recovery. Given their low cost and minimal risk, teaching patients simple lymphatic techniques or integrating KT periodically could serve as a useful adjunct to standard care. Moving forward, research should continue to define the ideal timing, patient population, and pairing of therapies to optimize outcomes.

Limitations

Several limitations should be considered in this review. First, many studies had small sample sizes, which may limit the generalizability of their findings. Additionally, there was significant variability in study methodologies, including differences in treatment timing, frequency, and follow-up duration, which complicates comparisons across studies. The use of subjective pain and satisfaction measures, such as self-reported scales, could introduce bias, and future studies could benefit from including more objective outcome measures.

Moreover, many studies did not control for confounding factors, such as patient comorbidities or concurrent therapies, which could impact results. Lastly, the heterogeneity of patient populations, including variations in age and health conditions, limits the applicability of findings to broader groups. Future research should aim for larger, more homogeneous samples, with standardized methodologies and a focus on objective outcomes.

Manual treatment emerges as a viable intervention that warrants further research for the following reasons. While most studies suggest positive outcomes, there is a need for more comprehensive and rigorous research to establish the efficacy of manual therapy across a broader range of conditions. With the growing interest in integrative and complementary therapies, investigating manual treatment can provide valuable insights into its role within the broader healthcare landscape. Moreover, understanding the mechanisms underlying manual therapy’s effects can help refine its application and identify the most suitable patient populations. The comprehensive literature review suggests that manual therapy provides some advantages, as opposed to yielding no benefits. Nevertheless, it is evident that further research is needed to substantiate these findings.

Table 3 presents the results of the risk of bias assessment for each included RCT, showing how the studies were judged across the five Cochrane RoB 2 domains, and providing an overall risk of bias rating that reflects the methodological quality of the evidence base.

**Table 3:** Risk of Bias Assessment Risk of bias was assessed independently by two reviewers using the Cochrane Risk of Bias Tool for Randomized Trials. The five domains evaluated included (1) bias arising from the randomization process, (2) bias due to deviations from intended interventions, (3) bias due to missing outcome data, (4) bias in measurement of the outcome, and (5) bias in selection of the reported result. Each domain was rated as low risk, some concerns, or high risk, with an overall judgment assigned to each trial. Abbreviations: RCT, Randomized Controlled Trial; VAS, Visual Analog Scale; NPRS, Numeric Pain Rating Scale; IM, Intramuscular; NRS, Numeric Rating Scale

Study (Author, Year)	Randomization Process	Deviations From Intended Interventions	Missing Outcome Data	Measurement of Outcome	Selection of Reported Results	Overall Risk of Bias
Karaborklu Argut et al. (2021) [24]	Low (RCT, adequate reporting)	Low	Low	Low (validated scale, NPRS)	Some concerns (limited detail on pre-specification)	Some concerns
Pichonnaz et al. (2016) [21]	Low	Low	Low	Low (VAS, blinded assessor)	Some concerns	Some concerns
Ebert et al. (2013) [25]	Low	Low	Low	Low (NPRS, standardized)	Some concerns	Some concerns
Licciardone et al. (2004) [26]	Some concerns (randomization described but with limited detail)	Low	Low	Low (medication dosage outcomes objective)	Some concerns	Some concerns
Jarski et al. (2000) [27]	Some concerns (older study, randomization less clearly reported)	Low	Low	Low (objective IM analgesic use)	Some concerns	Some concerns
Guney-Deniz et al. (2023) [28]	Low	Low	Low	Low (VAS, standardized)	Some concerns	Some concerns
Fujiura et al. (2020) [29]	Low	Low	Low	Low (VAS, with specific timing)	Some concerns	Some concerns
Vergili et al. (2022) [30]	Low	Low	Low	Low (VAS, standardized)	Some concerns	Some concerns
Cihan et al. (2021) [31]	Low	Low	Low	Low (VAS, standardized)	Some concerns	Some concerns
Tornatore et al. (2020) [32]	Low	Low	Low	Low (NRS, validated)	Some concerns	Some concerns

## Conclusions

In summary, manual therapy, including interventions such as kinesiotaping, HVLA techniques, OMT, MLD, and myofascial release, represents a viable adjunctive treatment that does not require specialized equipment and can be tailored to patient needs and provider expertise. The reviewed literature suggests that these modalities offer benefits compared to no treatment, particularly in pain reduction and functional improvement following TKR, although the strength of evidence varies by technique. While existing research supports manual therapy as a complementary strategy in postoperative rehabilitation, more comprehensive investigations are needed to compare modalities directly and establish standardized treatment protocols. Future studies, including meta-analyses and trials specific to postoperative manual interventions, are recommended to provide clearer insights.
